# Putative stem cells in the hemolymph and in the intestinal submucosa of the solitary ascidian *Styela plicata*

**DOI:** 10.1186/s13227-019-0144-3

**Published:** 2019-11-25

**Authors:** Juan Jiménez-Merino, Isadora Santos de Abreu, Laurel S. Hiebert, Silvana Allodi, Stefano Tiozzo, Cintia M. De Barros, Federico D. Brown

**Affiliations:** 1 Departamento de Zoologia, Instituto de Biociências, Universidade de São Paulo, Rua do Matão, Trav. 14, São Paulo, SP 101 05508-090 Brazil; 2 Centro de Biologia Marinha (CEBIMar), Universidade de São Paulo, São Paulo, Brazil; 3 Laboratório de Neurobiologia Comparativa e do Desenvolvimento, Pós-Graduação em Ciências Biológicas-Fisiologia, Instituto de Biofísica Carlos Chagas Filho, Universidade Federal do Rio de Janeiro, UFRJ, Rio de Janeiro, RJ Brazil; 4 Pós-Graduação em Ciências Morfológicas, Instituto de Ciências Biomédicas, Universidade Federal do Rio de Janeiro, UFRJ, Rio de Janeiro, RJ Brazil; 5 CNRS, Laboratoire de Biologie du Développement de Villefranche-sur-mer (LBDV), Sorbonne Universités, 06230 Paris, France; 6 Laboratório Integrado de Morfologia, Núcleo em Ecologia e Desenvolvimento Sócio Ambiental de Macaé, NUPEM, Universidade Federal do Rio de Janeiro, UFRJ, Macae, RJ Brazil; 7 Instituto Nacional de Ciência e Tecnologia em Estudos Interdisciplinares e Transdisciplinares em Ecologia e Evolução (IN-TREE), Salvador, BA Brazil

**Keywords:** Cell progenitors, Hemocytes, Intestine, Stem cells, Tunicates

## Abstract

**Background:**

In various ascidian species, circulating stem cells have been documented to be involved in asexual reproduction and whole-body regeneration. Studies of these cell population(s) are mainly restricted to colonial species. Here, we investigate the occurrence of circulating stem cells in the solitary *Styela plicata,* a member of the Styelidae, a family with at least two independent origins of coloniality.

**Results:**

Using flow cytometry, we characterized a population of circulating putative stem cells (CPSCs) in *S. plicata* and determined two gates likely enriched with CPSCs based on morphology and aldehyde dehydrogenase (ALDH) activity. We found an ALDH + cell population with low granularity, suggesting a stem-like state. In an attempt to uncover putative CPSCs niches in *S*. *plicata*, we performed a histological survey for hemoblast-like cells, followed by immunohistochemistry with stem cell and proliferation markers. The intestinal submucosa (IS) showed high cellular proliferation levels and high frequency of undifferentiated cells and histological and ultrastructural analyses revealed the presence of hemoblast aggregations in the IS suggesting a possible niche. Finally, we document the first ontogenetic appearance of distinct metamorphic circulatory mesenchyme cells, which precedes the emergence of juvenile hemocytes.

**Conclusions:**

We find CPSCs in the hemolymph of the solitary ascidian *Styela plicata*, presumably involved in the regenerative capacity of this species. The presence of proliferating and undifferentiated mesenchymal cells suggests IS as a possible niche.

## Introduction

Most metazoans possess migratory cells within their bodies, referred to in coelomate animals as coelomocytes and/or hemocytes [1]. The diversity in structure and function of these cells is considerable. However, they accomplish similar functions among taxa, namely, coagulation, immunological defense, oxygen transport, and tissue repair [2, 3]. It is widely accepted that animal hemocyte lineages derive from an evolutionarily conserved cell type, the hematopoietic stem cell (also blood stem cell) [1, 4, 5]. While hematopoietic stem cells are known to give rise to several hemocyte types in many animals [1], there are some animal taxa in which stem cells circulating in the hemolymph can also contribute to the formation of new animal bodies via asexual reproduction. Such is the case in many colonial invertebrates, such as colonial ascidians (Tunicata). In colonial ascidians, which display a modular layout formed by clonally related zooids that arise from asexual propagation [6], circulating progenitor cells—referred to as *hemoblasts* [5]—are known to be involved in whole-body regeneration and budding [7, 8].

The origin and function of circulating putative stem cells (CPSCs) have been described in ascidians of the subfamily Botryllinae (suborder: Stolidobranchia). In this group, multipotent cells in the hemolymph are progenitors of somatic tissues [9] and germline [10]. Botryllid CPSCs play a fundamental role in the biogenesis of new zooids [11–13], and in the transmission of germline and somatic cell lineages [12, 14]. In addition, the differentiation potential of hemoblasts has been experimentally proven in colonies surgically reduced to the peripheral tunic with circulating vessels containing hemolymph [15–17]. In these conditions, hemoblasts adhere to vessel walls and give rise to budlets, which mature into functional zooids [18, 19]. The cellular mechanisms that underlie budding have also been examined in one species in the suborder Phlebobranchia, *Perophora viridis* [17]. In both *P. viridis* and *B. schlosseri*, populations of self-renewing circulating progenitor cells seem to be involved in regeneration and budding [12, 17]. Whereas *Perophora* progenitors were proven to be pluripotent [17], circulating *Botryllus* progenitors are considered multi- or unipotent [11, 20].

*P. viridis* and *B. schlosseri* belong to separate suborders, within which coloniality evolved independently from solitary ancestors [21]. In fact, coloniality has originated multiple times within the ascidians [6, 11, 21]. Thus, the function and presence of CPSCs in asexual development have plausibly convergently evolved across the ascidians. It is likely that the origin of the CPSC cell type underlies the emergence of coloniality in some ascidian lineages. However, the function and presence of such CPSCs in solitary species are unclear. Therefore, investigating the ancestral character state of hemoblasts in a solitary species may provide insights into the events giving rise to CPSCs and coloniality.

The characterization of hemocytes in ascidians has historically lacked a uniform nomenclature due to distinct identification protocols or personal biases in selecting the most important characters for identification [5, 16, 22]. In Styelidae, coloniality has evolved at least twice [21] and hemocytes of colonial species in this family have been previously described [23–25]. Therefore, we focus this study on the solitary *Styela plicata* as an outgroup species to approximate the ancestral character state of hemoblast populations in the styelid ascidians. *S. plicata* exhibits five main cell types in the hemolymph: granulocytes, lymphocyte-like cells, morula cells, pigment cells, and hemoblasts [25, 26] (Table 1). In *S. plicata*, hemoblasts are presumably involved in regeneration, including neural regeneration, and share similar morphological characteristics to the colonial circulating stem cells, including a uniform round shape, a relatively small size (5 μm in diameter), a high nuclear⁄cytoplasmic ratio, and prominent nucleoli [25–30]. Thus, a somatic stem cell role has been proposed for this specific subpopulation of hemocytes.

**Table 1:** Hemocyte types identified in *Styela plicata*

Cell type	Diameter (µm)	Identified content	Possible function	References
Granulocyte	4.2–5.2	Heparin and histamine	Similar to vertebrate basophiles	de Barros et al. [46]
Lymphocyte-like cell	3.1–4.8	Nitric oxide	Signaling in defense mechanisms	de Barros et al. [25]
Hemoblast	6.1	None	Regeneration	Medina et al. [27]; Wright [5]
Morula cell	8.8–16.1	Phenoloxidase	Cytotoxicity	Cammarata et al. [83]
Pigment cell	5.1–13.1	Nitrogenated compounds	Pigmentation	Wright [5]

Here, we report the occurrence of putative circulatory stem cells in the hemolymph of the solitary *S. plicata* by cytological observations and flow cytometry. Circulatory stem cells in the colonial styelid *B. schlosseri* have been shown to undergo dynamic migration patterns between transient niches of zooids and buds [23, 31]; however, studies of stem cell niches in solitary styelids remain scarce. In this study, we used cell morphometric parameters and a stem cell marker (i.e., ALDH) in combination with imaging flow cytometry to confirm the presence of CPSCs in the circulatory system of solitary *S. plicata*. High ALDH enzymatic activity occurs in progenitor cells of different animals, and has been used to isolate populations of cells enriched with CPSCs (germline and somatic stem cells) in colonial styelid ascidians, including *B. schlosseri* [12, 15, 32] and *Polyandrocarpa misakiensis* [33, 34]. Next, we identified a site that contained hemoblast aggregations suggesting the presence of a putative niche in the intestinal submucosa of *S. plicata* using light and electron microscopy. Lastly, we externally examined the time and place in development in which motile cell populations emerged. In contrast to the reduced movements observed in embryonically derived mesenchymal cells of the ascidian larva, motile cells in the hemocoel (i.e., hemocytes) first appeared during metamorphosis concomitantly to the expansion of an extracorporeal vasculature involved in settlement, but before the heart differentiated. These findings are discussed within an evolutionary framework to understand the original roles of CPSCs in solitary and colonial styelids.

## Methodology

### Animal collection

*Styela plicata* is a cosmopolitan species, abundant in harbors of the Atlantic coastline. Juveniles grow rapidly and can attain its maximum size of 8 cm in 6 months [35, 36]. In Brazil, this species is found along the southeastern coastline, including the harbors of Rio de Janeiro, São Sebastião, and Santos [35]. The life cycle encompasses a brief (1–2 days) larval period before the animal settles on a definitive substrate. For this study, adult individuals were collected from the Ilhabela Yacht Club, São Sebastião (23°46′20″ S, 45°21′20″ W), the Ponte Edgard Perdigão dock, Santos, (23°59′30.60″ S, 46°18′10.27″ W), and Praia da Urca harbor (22°56′43″ S, 043°09′48″ W), Rio de Janeiro, Brazil according to the guidelines approved by SISBIO/IBAMA (no 14689). Animals were maintained in aerated aquaria containing seawater and following local temperature conditions that ranged between 20 and 24 °C, pH between 8 and 8.5, and salinity 35 ppt [37].

### Hemocyte description

To harvest hemocytes, five adult *S. plicata* were cut through the oral siphon and hemolymph was decanted into a 1.5-mL tube half-filled with *Botryllus* buffer (25-mM HEPES, 10-mM cysteine, 50-mM ethylenediaminetetraacetic acid in seawater, pH 7.5). The hemocyte suspension was centrifuged at 780×*g* for 10 min, and cells were resuspended in 60-µL *Botryllus* buffer. Drops of this suspension were transferred onto SuperFrost plus slides (Fisher, Waltham, MA, USA), and left to settle for 30 min. To distinguish intracellular compartments, hemocytes were stained with saturated Neutral Red solution (10 mg/mL) for acid vesicles, or Sudan Black staining for lipids. Neutral Red (Merck, Darmstadt, Germany) saturated solution in filtered sea water (FSW) was added in equal volume to the slides and cells were immediately prepared for observation. For Sudan Black staining, hemocytes were fixed in 70% ethanol, immersed in a Sudan Black (Sigma Aldrich, St. Louis, USA) saturated solution (70%), washed once in ethanol (3 min), and once in distilled water (3 min). Coverslips were mounted with glycerol and sealed with nail polish.

Five cell types comprise the hemocyte population in *S. plicata*: hemoblasts, lymphocyte-like cells, granulocytes, morula cells and pigment cells [25, 26] (Table 1). To classify and measure relative frequencies of cell types, including intermediate states, we defined and described general morphotypes that can be identified with light microscopy. The relative frequency of morphotypes was estimated by counting Neutral Red-stained cells (two replicates for each individual). Relative frequencies were evaluated after counting 10 fields for each glass slide, corresponding to at least 400 cells, from contiguous fields of view of the cell suspension at 63×. We used four adult individuals of similar size (approximately 4 cm in length) as replicates for our analyses.

### Flow cytometry

Twelve adult animals with a wide range of sizes (1–6.5 cm, 0.6–14 g wet mass), to capture variation between body mass, were selected for analyses. Water ejection was induced through light pressure and wet weight was measured. Hemolymph was extracted from each individual as previously described. Hemocyte concentrations were calculated using a hemocytometer. All samples cell concentrations were above 3 × 10^6^ cells/mL. We used 50-µL hemocyte suspensions at a concentration of approximately 1 × 10^6^ cells/mL, which were acquired by centrifugation at 790×*g* for 10 min and resuspension in *Botryllus* buffer.

To further identify CPSC populations in the hemolymph, we used a combined strategy for cell sorting that evaluates light refraction and enzymatic features to separate populations of hemocytes in these individuals. We used the viable stem cell, BODIPY-aminoacetaldehyde (BAAA)-based kit, ALDEFLUOR^TM^ (STEMCELL Technologies) to test for the presence of undifferentiated cells. ALDEFLUOR^TM^ has been extensively used to detect hematopoietic stem cells and progenitors in humans and mice [38, 39, 40]. Additionally, ALDH is present both in mammalian stem cells [41] and in the growing asexual buds of a colonial ascidian, *Polyandrocarpa misakiensis* [33, 34] as well as somatic and germline circulating stem cells in *B. schlosseri* [12, 32]. Cells were stained in 1–2-mM BAAA solution in *Botryllus* buffer at 37 °C for 20 min. *N*,*N*-diethylaminobenzaldehyde (DEAB), an inhibitor of ALDH, was used at 200 mM in the staining solution to generate negative controls. Cells were washed twice, resuspended in Botryllus buffer and maintained on ice until analysis.

Sample acquisition was performed with an imaging flow cytometer (FlowSight, Amnis-Merck Millipore) from the Central de Aquisição de Imagens e Microscopia of the Instituto de Biociências (CAIMI-IB). Acquisition speed was set to low and photos were captured at the highest resolution. About 20,000 cells were acquired based on area and the aspect ratio, defined as the value of minor axis divided by major cell axis on channel 1. The focused cells were gated using root mean squared gradient (RMS gradient) based on channel 1. Channels 1, 2, and 6 were used to analyze brightfield, ALDEFLUOR^TM^-labeling, and side scatter (SS) parameters, respectively, using the IDEAS software (Amnis-Merck Millipore). Cell doublets and debris were excluded from the single cells used in the analyses based on area and aspect ratio features (Additional file 1: Figure S1). ALDH + cells and subpopulations of hemocytes were determined based on channel 2 (green) and channel 6 intensities. No autofluorescence was detected in *S. plicata* hemocytes (Additional file 1: Figure S1). Data acquisition was performed using the IDEAS package (Amnis, Seattle, USA). To detect significant increases in ALDH + event abundances using the ALDEFLUOR^TM^ test, we performed Wilcoxon matched-pairs signed rank tests between tests and controls (as defined by the ALDH inhibitor DEAB).

### Whole-body histological analyses

Whole animals were fixed in 4% paraformaldehyde (PFA) in FSW for 24 h and then embedded in paraffin. Whole individuals were cut into sagittal sections of 6 μm using a Leica RM2255 microtome (Wetzlar, Germany). For observing general tissue morphology and organization, we used hematoxylin and eosin staining following the method of Gurr [42]. Mallory–Cason trichrome and Gomori trichrome stains were applied following Humason’s protocol [43].

### Histology

The intestines of five ascidians were dissected and fixed with 4% PFA in artificial seawater for 24 h. Tissues were dehydrated in increasing concentrations of ethanol (70%, 80%, 90%, 100%) for 30 min in each concentration. The tissues were then cleared with xylene for 20 min, and embedded in Paraplast (Sigma, St. Louis, MO, EUA). Histological sections of 5 µm were obtained on a rotating microtome (American Optical). Tissue sections were dewaxed with xylene and rehydrated in decreasing concentrations of ethanol and water.

### PIWI labeling

Histological sections obtained as described above were incubated with 10-mM citrate buffer (with 0.05% Tween 20, pH 6) at 90 °C for 30 min for antigen retrieval, and cooled for 20 min in a 0.1% Tween 20 (PBT) solution in 1× phosphate-buffered saline (PBS), followed by a 5-min wash with PBT. Sections were incubated with rabbit polyclonal primary antibody against PIWI (1:100; Abcam ab5207, Cambridge, UK) in PBT for 48 h (Additional file 3: Figure S2). Then, the sections were washed in PBS, incubated with anti-rabbit biotin (K0679, DAKO; 1:100) for 1 h, subsequently incubated in Cy3-associated streptavidin (Sigma-S6402; 1:500) for 1 h. After, the sections were washed in PBS, the nuclei were labeled with DAPI for 10 min and the sections washed and mounted with Fluoromount (Sigma-F4680). We included sections without the primary antibody incubation as negative controls. We found no nonspecific labeling in the controls.

We analyzed 10 randomly selected images from histological sections of two animals (*n* = 2). Percentages of positive nuclei were obtained independently for each intestinal layer, i.e., mucosa, submucosa, and serosa. Percentages were then compared using Student’s *t* test. Results were expressed as the mean ± standard error, and differences were considered statistically significant at *p* < 0.05 (*). The analyses were done using GraphPad Prism version 6.00 (GraphPad Software, Inc.).

### pHH3 labeling

Sections obtained as outlined above were kept in 2-M HCl at 50 °C for 30 min, then transferred to 10-mM citrate buffer pH6 at 90 °C for 30 min, chilled in PBT for 20 min and incubated in anti pHH3 rabbit polyclonal antibody (Santa Cruz, sc8656-R; 1:50) for 4 days at 4 °C. The final PBS washes, incubation with secondary antibody using the labeled streptavidin–biotin, DAPI staining, and mounting were performed as described above.

Data were collected from eight randomly selected histological sections of the intestines of two animals (*n* = 2). For comparison, immunofluorescence was also performed in histological sections of the epidermis. The percentages of positive nuclei were obtained independently for each intestinal layer, i.e., mucosa, submucosa, and serosa, as well as for epidermis tissue. The percentages were then compared as described above.

### Transmission electron microscopy

Ascidian intestines were dissected and fixed with a 2.5% glutaraldehyde solution and 0.1-M sodium cacodylate in artificial sea water for 24 h, washed in 0.1-M cacodylate buffer and post fixed in OsO_4_ for 40 min. The material was then pre-contrasted with 1% uranyl acetate diluted in artificial sea water for 24 h. The intestines were washed again in 0.1-M cacodylate buffer, dehydrated in increasing concentrations of acetone (30% to 100%) and infiltrated in Embed 812 resin (Electron Microscopy Sciences). Ultrathin sections (70 nm) were obtained with an RMC ultramicrotome, collected on copper grids (300 mesh), contrasted with 2% uranyl acetate for 20 min and with 1% lead citrate for 3 min. The sections were observed under a JEOL 1011 transmission electron microscope, operated at 80 kV, in the Rudolf Barth Electron Microscopy Platform from Instituto Oswaldo Cruz/FIOCRUZ (Rio de Janeiro).

### Fertilization and rearing in the laboratory

In vitro fertilization and culture were modified from Swalla [44]. One–two days after collection, sets of 4 mature adults were separated and each specimen was bisected on the sagittal plane. Gonads were separated and removed from both halves with forceps, and minced over a mesh (250-μm pore size) suspended over a beaker with 50 mL of FSW. The resulting gamete suspension was kept still for 15 min, after which all oocytes settled on the bottom of the beaker. Supernatant containing suspended sperm was decanted into 15-mL tubes for cross-fertilization. The remaining oocyte medium was washed via reverse filtration with a 50-μm mesh and resuspended in FSW. Sperm from each individual was pooled to cross-fertilize each oocyte set. Gametes were mixed for 15 min before removal of excess sperm. Further washes were performed through resuspension in FSW every 30 min for the first 2 h after fertilization, and three more times every hour.

At 12 h post-fertilization, larvae were left in the dark to settle on glass dishes. At around 48 h post-fertilization, after tail resorption, young metamorphs were carefully removed from the glass with a tungsten needle and relocated to microscopy slides using a glass pipette. During settlement, adhesive papillae fully differentiated, which allowed the animals to adhere to the slide surface. Development was documented with a stereomicroscope Leica M205 FA and an inverted microscope Leica DMi8.

## Results

### Frequency and characterization of hemocyte morphotypes in *Styela plicata*

**Fig. 1:**
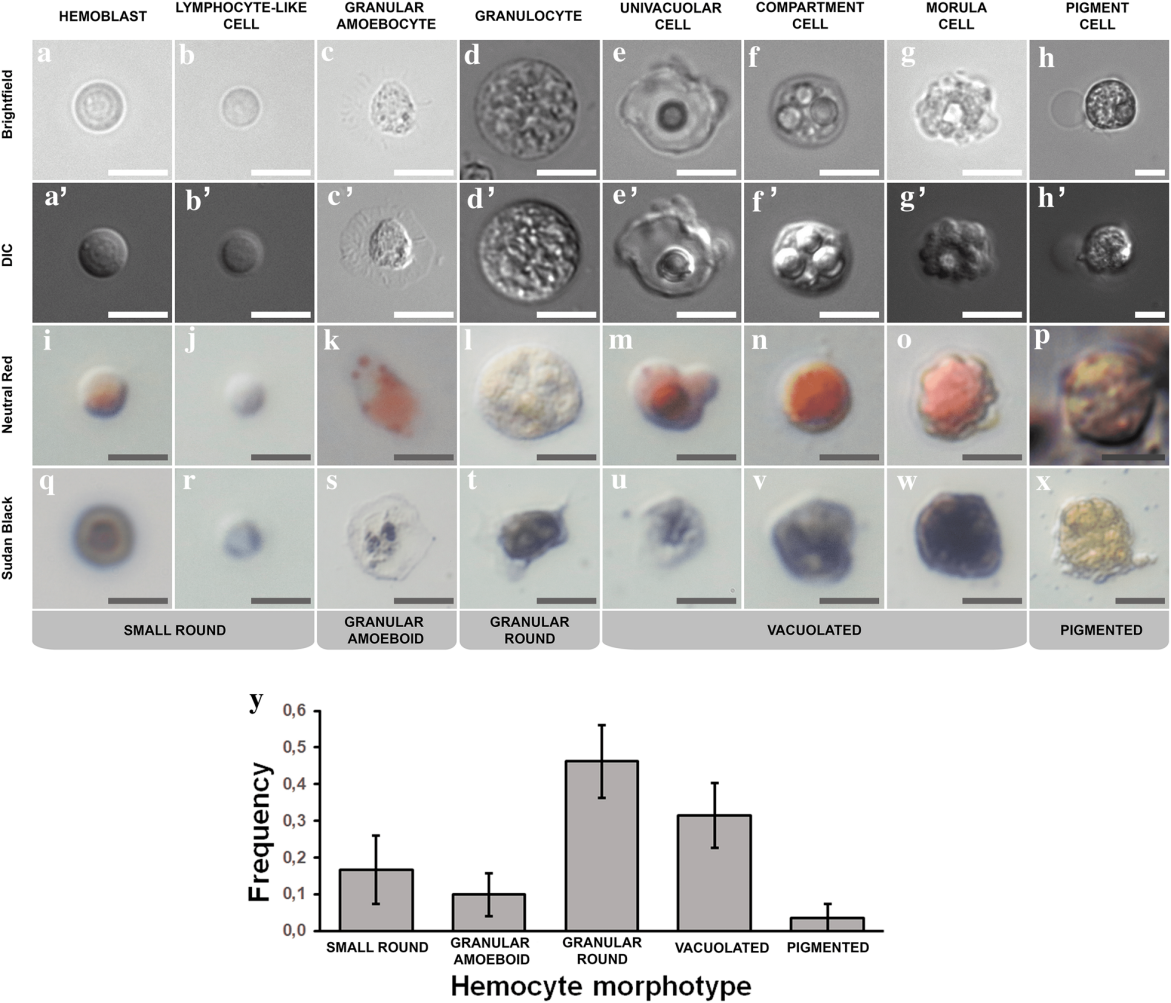
*Styela plicata* hemocytes (**a**–**x**) and frequencies (**y**)**. a**, **a′**, **i**, **q** Hemoblast, one of the two types of *small round* cell with big nucleus (see text for explanation); **b**, **b′ j**, **r** lymphocyte-like cell, the other *small round* cell with big nucleus that is generally smaller than the hemoblast (**a**, **a′**, **i**, **q**); **c**, **c′**, **k**, **s** granular amoebocyte, includes *granular ameboid* cells with characteristic scattered granules and an irregular membrane; **d**, **d′**, **l**, **t** univacuolar refractile granulocyte, large *granular round* cells; **e**, **e′**, **m u** univacuolar cell, *vacuolated* cell type with one conspicuous vacuole; **f**, **f′**, **n**, **v** compartment cell, includes *vacuolated* cells with 3–5 vacuoles; **g**, **g′**, **o**, **w** morula cell, *vacuolated* cells with numerous vacuoles; **h**, **h′**, **p**, **x** pigment Cell, includes all *pigmented* cell types; **y** relative frequencies of cell morphotypes show *granular round* cells as most abundant, whereas *pigmented* cells are least abundant; *small round* cells (hemoblast and lymphocyte-like cells) represent about a fifth of all cell types (*n* = 4 individuals, approx. 300 images analyzed). **a**–**h** Brightfield, **a′**–**h′** DIC, (**i**–**p**) Neutral Red, (**q**–**x**) Sudan Black. Hemocyte types (shown above) are based on de Leo [22] and de Barros [36, 45], and morphotypes used to calculate frequencies in (**y**) and in this study are shown below in gray tags. Scale bars = 5 µm

We established hemocyte categories based on morphological features. We found eight cell morphotypes that were previously described in *S. plicata*, as well as in other ascidians [5, 22, 25, 45, 46]: lymphocyte-like cells, hemoblasts, univacuolar refractile granulocytes, amoeboid granulocytes, compartment cells, univacuolar cells, morula cells, and pigment cells (Fig. 1a–x). Roughly 50% of hemocytes were not easily classified into any of the previous cell types (data not shown). For simplicity, we decided to combine some of the hemocyte categories above into the following five hemocyte morphotypes (Table 2):

**Table 2:** Cell morphotypes used in this study to classify total hemocyte populations in *Styela plicata*

Cell morphotype	Diameter (µm)	Percentage (%)	Possible types	Description
Small-round cells	2.8–6	16.7 ± 9.3	Lymphocyte-like cells, hemoblasts	Circular cells with a high nucleus/cytoplasm ratio. Granules scarce or absent
Granular amoeboid cells	4–8	9.9 ± 5.9	Granulocytes and amoeboid cells in differentiation	Amoeboid cytoplasm with granular contents
Large granulocytes	5–8.6	46.2 ± 9.9	Fully differentiated granulocytes, univacuolar refractile granulocytes	Rounded contour, numerous granular structures in cytoplasm, a large refractile vacuole may be present
Vacuolated cells	5–20	31.4 ± 8.8	Simple univacuolar cells, compartment cells, morula cells	Cells with one or more prominent basophilic vacuoles in the cytoplasm
Pigmented cell	8–13	3.5 ± 3.9	Pigment cell	Large cells, cytoplasm saturated with compartments filled with amber pigment

1. Small round cells: These represent the smallest cell types (2.8–6 µm in diameter), which represent 1/6 of all cells in the hemolymph (16.7 ± 9.3%, *n* = 1384). Circular cells with a high nucleus/cytoplasm ratio with few or no granules in the cytoplasm.
2. Granular amoeboid cells: Small amoeboid cells (4–8 µm) with granular cytoplasm that represent 1/10 of all cell types (9.9 ± 5.9%, *n* = 1384).
3. Fully differentiated granulocytes: Rounded cells with intermediate size (5–8.6 µm) that represent nearly 1/2 of all hemocytes (46.2 ± 9.9%, *n* = 1384). These cells contain numerous granular structures in the cytoplasm, which are most likely inside a large refractile vacuole.
4. Vacuolated cells: These cells represent 1/3 of all cells (31.4 ± 8.8%, *n* = 1384), and vary in size (5–20 µm). One or more prominent basophilic vacuoles are present in the cytoplasm.
5. Pigment cells: These large cells (8–13 µm) are rare (3.5 ± 3.9%, *n* = 1384), and are saturated with cytoplasmic compartments filled with amber pigment.

Overall, granulocytes represented the most common morphotype, followed by vacuolated cells, small round cells, and pigmented cells (Fig. 1y; Table 2). Hemocyte populations exhibited different frequencies between individual adults.

### Hemocytes with high aldehyde dehydrogenase activity were found in the hemolymph of *Styela plicata*

**Fig. 2:**
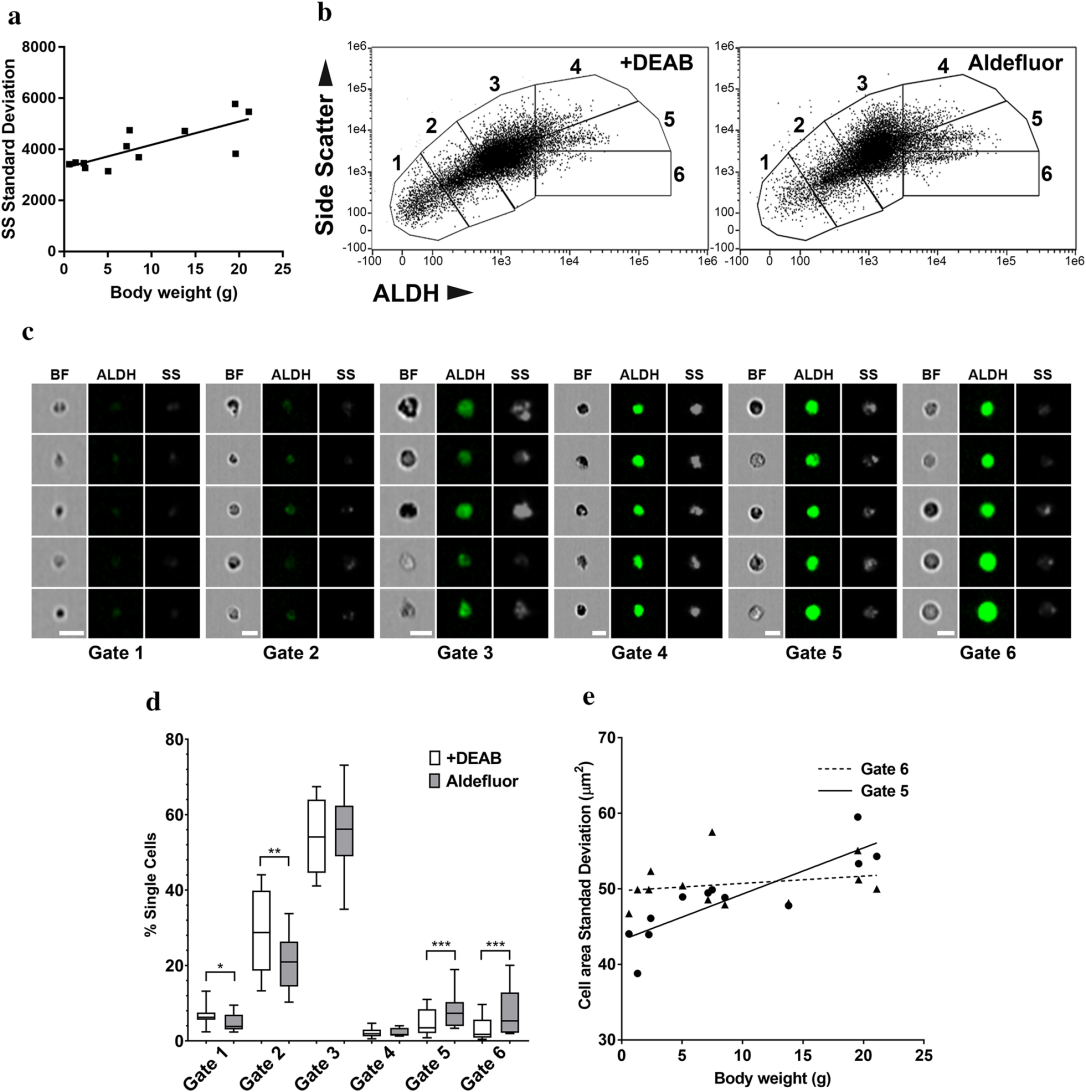
Imaging flow cytometry analysis of hemocyte populations in *Styela plicata*. **a** Cell complexity of total hemocytes is shown (Side Scatter (SS) Standard deviation) as a function of body weight (*r*^2^ = 0.58, *p* = 0.038; *y* = 172.3*x* + 5078); **b** gating strategy applied to SS/ALDH scatterplots of one representative ascidian specimen (gates 1–6); Gates 5 and 6 show an increase in cells in ALDEFLUOR^TM^-treated samples as compared to the ALDH inhibitor control (+DEAB); **c** gallery of images from selected gates representing cell morphologies, showing brightfield (BF), ALDH, and SS channels; **d** cell percentages corresponding to each gate on ALDEFLUOR^TM^ test samples (gray) vs. controls (white). Note the increase of cells in gates 5 and 6 (***p < 0.01) and the decrease in cells in gates 1 (*p < 0.05) and gate 2 (**p < 0.025), asterisks indicate significant differences using Wilcoxon matched-pairs signed rank tests; **e** cell complexity of hemocytes in gates 5 and 6 is shown (cell area standard deviation) as a function of body weight, gate 5 regression: *r*^2^ = 0.73, *p* = 0.0004, *y* = 0.61*x* + 43.21, gate 6 regression: *r*^2^ = 0.055, *p* = 0.59, *y* = 0.0962*x* + 49.77. Note that cells in gate 6 do not change in cell complexity in ascidians of different size

To analyze hemocytes present in the hemolymph of *S. plicata*, we used imaging flow cytometry to characterize morphological features such as cell size, complexity (measured through side and forward scatter, respectively), and ALDEFLUOR^TM^-generated fluorescence (Fig. 2). We first wanted to analyze the inter-individual variation among several adult *S. plicata* specimens. Interestingly, we observed a positive correlation (*r*^2^ = 0.58, *p* = 0.038) between the size of individuals (measured by mass/weight) and the level of variation in cell complexity of hemocytes in the hemolymph (measured by the standard deviation of the SS of each hemolymph sample) (Fig. 2a). Next, we analyzed CPSCs in the hemolymph by the use of a viable cell-permeable fluorescent aldehyde dehydrogenase (ALDH) substrate, BODIPY aminoacetaldehyde (BAAA) (Fig. 2b–e). Hemocyte scatter plots relating cell complexity (SS) and aldefluor-generated fluorescence (ALDH activity) showed minimal variation across individual samples; this allowed us to consistently use the same gating system (gates 1–6) to include most cells in the scatter plot across the twelve individual samples (Fig. 2b–e). Coordinates of the selected gate polygons (gates 1–6) are provided in Additional file 2: Table S1. Five randomly selected cells for each gate are shown to highlight the variation of cell types we observed by imaging flow cytometry analyses (Fig. 2c). While cells in each gate varied notably in size, shape, and morphology (brightfield, BF), the levels of fluorescence (ALDH enzymatic activity) and cell complexity (SS) assisted us in the assignment of the distinct informative regions (Fig. 2b, c). Gates 1, 2, and 3 showed distinct dispersion patterns and increasing degrees of complexity, as well as BAAA fluorescence (Fig. 2b, c). Gates 4, 5, and 6 indicated high ALDH activity and were considered above the threshold established for positive ALDH + cells, i.e., above relative intensity values of 3100 (Fig. 2b). When comparing all gates, we consistently observed higher numbers of cells in gates 5 and 6 (i.e., the gates corresponding to high ALDH activity): these two gates contained 7.36% (± 1.96) more cells than in the corresponding gates of the DEAB control (Fig. 2b, d). To test whether the cells in gates 5 and 6 (with high ALDH) were part of a single population of cells, we performed multiple correlation analyses between adult size and gate values of all recorded cell measurements. Only one population of cells (gate 6) showed little or no variation in cell size among individual adults of different size (Fig. 2e), suggestive of a homogenous population that may contain the CPSCs with hemoblast-like cell characteristics. In contrast, the other informative region with high ALDH + cells (gate 5) showed an increase in the standard deviation of hemocyte area in bigger ascidians (Fig. 2e), suggestive of a more heterogeneous population of cell types within this gate. Therefore, the population of cells at gate 6 meets all morphological criteria of CPSCs: small size, low granularity, and high ALDH activity (most ALDH + cells) suggesting an enrichment of CPSCs at this gate.

We reasoned that if CPSCs contain low cell complexity and present high ALDH activity as expected for other undifferentiated cell types, the ALDEFLUOR^TM^ assays would show relatively high numbers of cells in gates that show low SS and high ALDH (i.e., gates 5 and 6), and at the same time show relatively low numbers of cells in gates with low SS and low ALDH (i.e., gates 1 and 2). As predicted, when we compared the scatterplot of the ALDEFLUOR^TM^ assay showing ALDH + cells (Fig. 2b right) to the scatterplot of the basal background fluorescence control showing BAAA treated cells together with the ALDH inhibitor DEAB (Fig. 2b left), we observed a higher number of cells in gates 5 and 6 (i.e., low SS and high ALDH) in the ALDEFLUOR^TM^-treated sample than in the DEAB control (Fig. 2b and d), whereas cells in gates 1 and 2 (i.e., low SS and low ALDH) were significantly reduced in the ALDEFLUOR^TM^-treated sample than in the DEAB control (Fig. 2b and d). We interpret that many of the low complexity cells located in gates 1 and 2 of the ALDH inhibitor control (Fig. 2b left), correspond to the low complexity cells and high ALDH + cells that become localized in gates 5 and 6 of the ALDEFLUOR^TM^ assay (Fig. 2b right). Altogether, these data show that circulatory cells with low complexity and high ALDH activity are present in the *S. plicata* hemolymph, which provides evidence for the occurrence of CPSCs in the hemolymph of *S. plicata*.

### The intestine exhibits undifferentiated hemocyte clusters in the subepithelial region

**Fig. 3:**
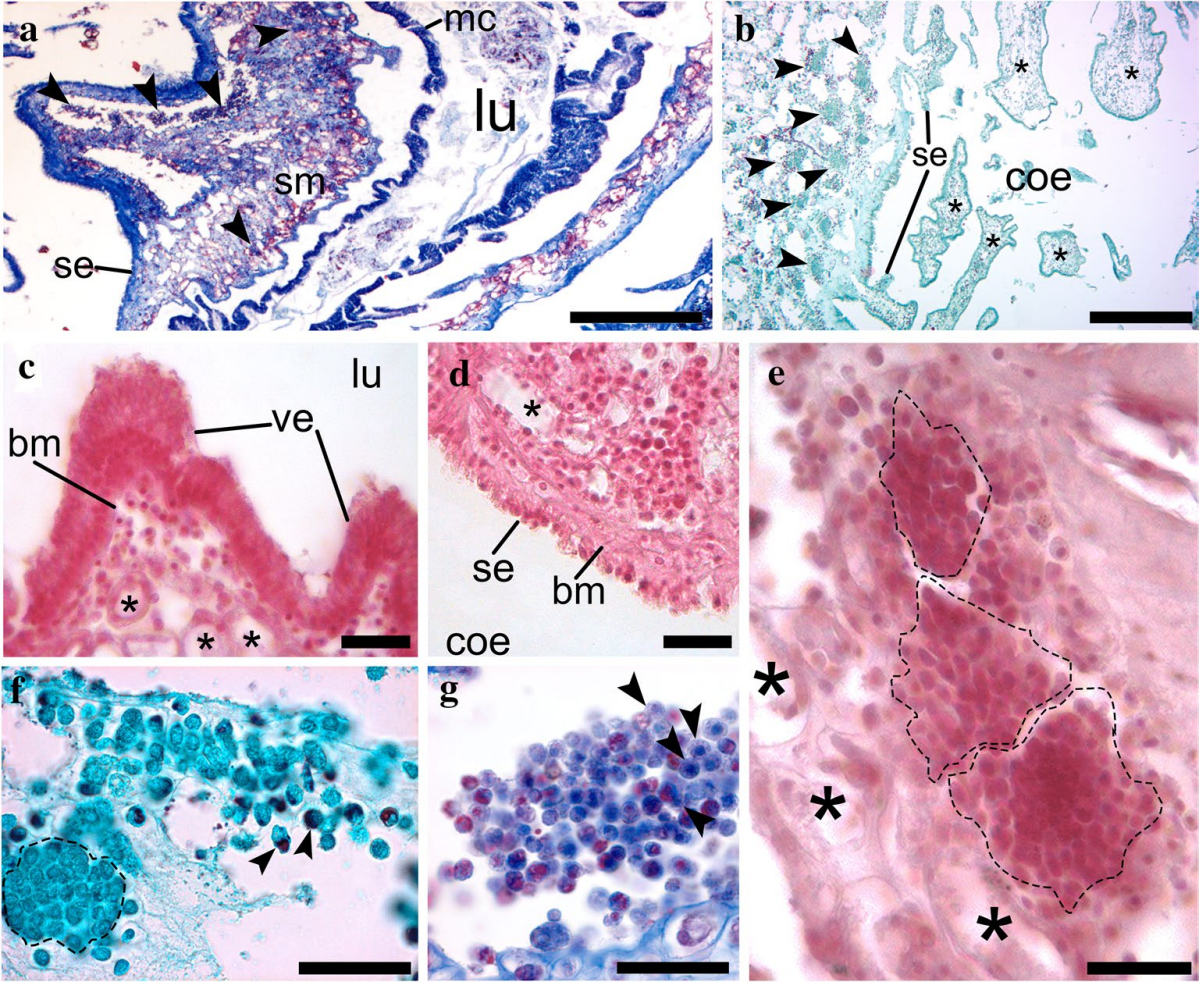
The cellular structure of *Styela plicata* intestine, with putative hemoblast clusters found in the submucosa. **a** Sagittal section of *Styela plicata* intestine showing its layers, clustered hemoblasts are indicated (arrowheads). **b** The interface of the serosa with the body cavity (coelom). Hemoblast clusters are indicated with arrowheads. Serosa projections are labeled. Asterisks mark projections of the serosa to the coelom. Note that the connective tissue contains hemoblast clusters. **c** Section of the mucosa, showing villi. Glandular structures can be seen beneath the epithelium (asterisks). **d** Section of the serosa. The asterisk marks a glandular structure. **e** Section of the submucosa. Hemoblast clusters are delineated (dashed line). Asterisks highlight glandular structures. **f** Detail of niches (delineated with dashed line) located at the outer submucosa. Arrowheads point to free cells with conspicuous granules. **g** Niche within the submucosa. Arrowheads point to characteristic hemoblasts. Staining techniques applied: Mallory–Cason trichrome (**a**, **g**), Hematoxylin and Eosin (**c**–**e**), Gomori trichrome (**b**, **f**). (mc) mucosa, (lu) lumen of the intestine, (bm) basement membrane, (ve) villus extension, (coe) coelom. Scale bars = 5 mm in **a**; 2 mm in **b**; 50 µm in **c**–**g**

In the intestine of *Styela plicata,* it is possible to identify three clearly distinct histological regions (Fig. 3). First, the most internal epithelium surrounding the lumen was constituted by a monolayer of columnar cells and a thin basement membrane, and is referred to here as the mucosa (Fig. 3a, c). Second, a middle region with connective tissue and glands, as well as compartmentalized cells (Fig. 3a, b, e–g) is hereinafter referred to as the submucosa. And third, an external region constituted by connective tissue and a simple cubic epithelium (Fig. 3a, b, d) that rests on the coelomic side and shares a common space with other organs through a series of epithelium-bound protrusions or thin layers of muscles is referred to here as the serosa layer (Fig. 3b).

**Fig. 4:**
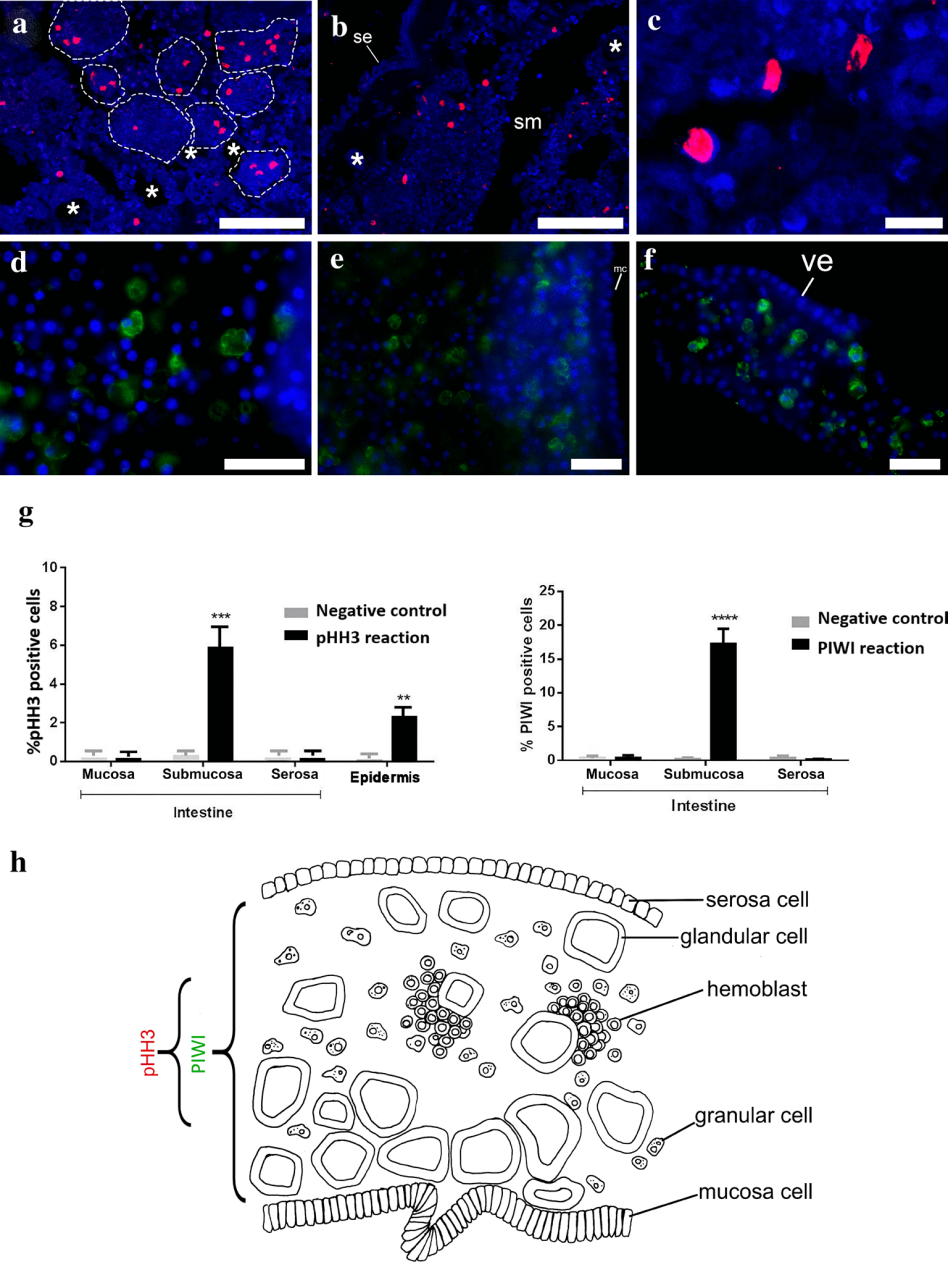
The intestine of *Styela plicata* presents PIWI + cells and pHH3 + cells. **a**–**c** Phosphohistone (pHH3) immunofluorescence shows antibody reaction in the region of the intestinal epithelia in *Styela plicata*. All cell nuclei were stained with DAPI (blue). **a** Wide view of the submucosa region, showing hemoblast aggregations (delineated by dotted lines), glandular structures are highlighted with asterisks. Labeled cells are pseudocolored red. **b** Wide view of the intestine subepithelial region below the serosa. Glandular structures are highlighted with asterisks. **c** Detail of labeled cells with pHH3 antibody in the submucosa. **d**–**f** Immunofluorescence showing antibody reaction with PIWI in the subepithelial region of *S. plicata* intestine. All cell nuclei were stained with the nucleus marker DAPI (blue) (**d**) Detail of the submucosa, labeled cells are pseudocolored green. **e** Detail of the subepithelial region. **f** Intestinal villi showing PIWI + cells at the subepithelial region. **g** Cell counts of pHH3 + cells in different regions of the intestine and the epidermis as an external control tissue (left) and PIWI (right) immunofluorescence in the intestine. Epidermis tissue was used for the control group. **h** Distribution of the serosa, submucosa, and mucosa cell types in a transverse section of the *S. plicata* gut showing the range of distribution of pHH3 (red) and PIWI (green) positive cells. (*p < 0.05). (se) serosa, (sm) submucosa, (m) mitochondria, (n) nucleolus. Scale bars = 100 µm in **a**, **b**; 20 µm in **c**–**f**

In the submucosa, we identified clusters of hemocytes that appeared to be hemoblasts (Fig. 3). These clusters showed positive expression of the mitotic marker phosphorylated histone 3 (pHH3) and the undifferentiation marker PIWI by immunofluorescence analyses (Fig. 4a). pHH3 + cells were observed in putative hemoblast clusters in proximity to glandular structures in the submucosa (Fig. 4b–d, h), but no positive cells were observed in the mucosa or the serosa (Fig. 4h). Although pHH3 + cells varied in frequency across intestinal regions and individuals, we observed at least twice as many proliferating cells in the submucosa (approx. 5.9% ± 1.9 *n* = 8) when compared to proliferating cells of the epidermis (approx. 2.3% ± 0.98 n = 3) (Fig. 4h). In addition we found a high incidence of PIWI expression (approx. 17.24% ± 5.9 *n* = 8) in scattered cells of the submucosa (Fig. 4d–f, i), but no expression in the mucosa or the serosa. PIWI was unevenly expressed in the cytoplasm of distinct cell types, including putative hemoblasts and granular cell types (Fig. 4e–g).

**Fig. 5:**
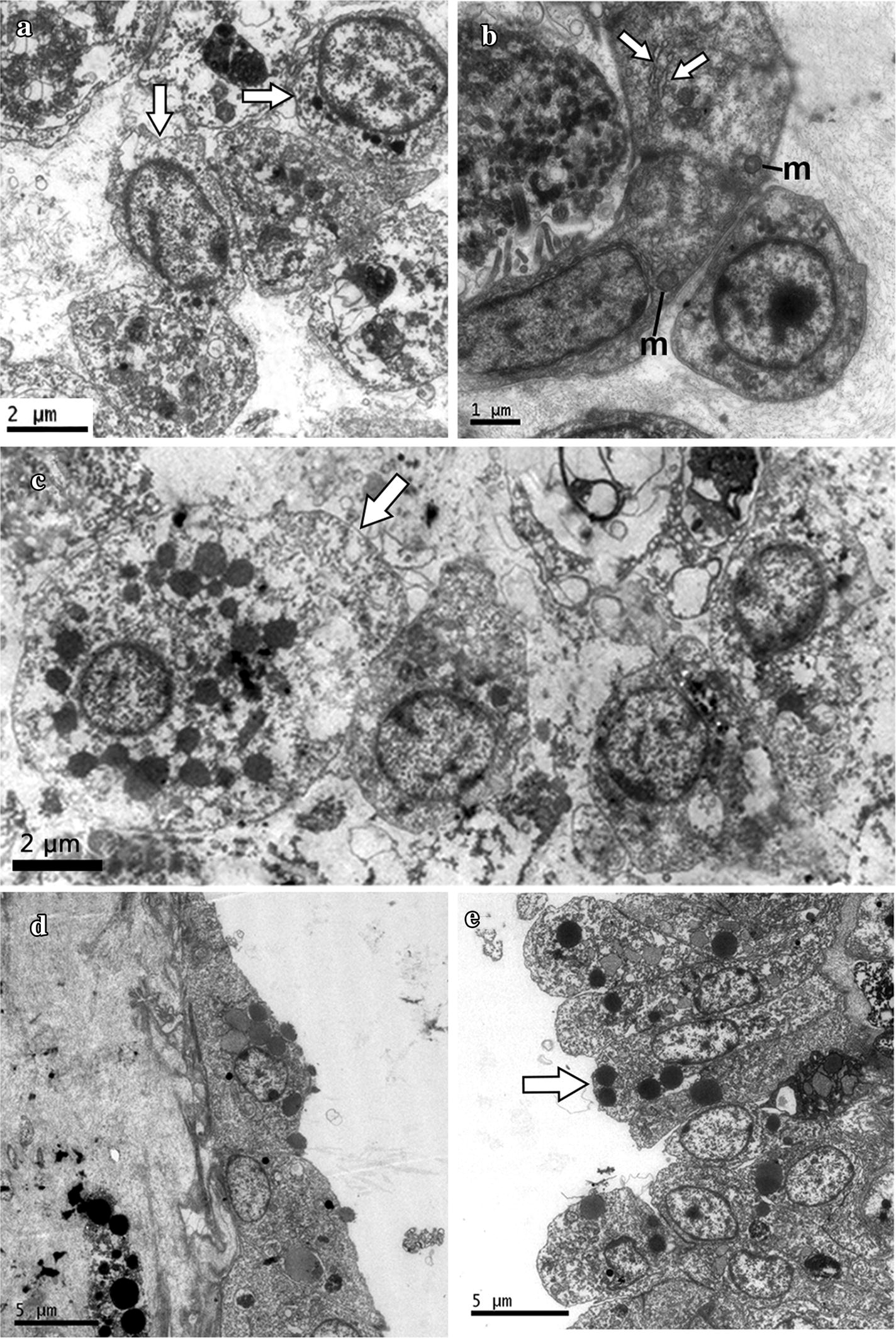
Ultrastructure of putative hemoblasts and other cell types in the *Styela plicata* intestinal submucosa. **a** Cell clusters in the submucosa include undifferentiated cells or putative hemoblasts, as well as other cell types in different stages of maturation. The two lateral cells (arrows) show features of undifferentiation or early stages of differentiation, including a euchromatic nuclei, and small cytoplasmic dense granules; whereas the cell in the center presents features of late differentiation, including larger dense vesicles in the cytoplasm and an irregular plasma membrane. **b** An elongated intestinal glandular cell type with endoplasmic reticulum (ER, white arrows) and mitochondria (m) is shown next to a putative hemoblast with a large nucleus and distinct nucleolus. **c** Cells at different stages of maturation in the submucosa; one granulocyte is shown (arrow) next to three smaller cells with irregular plasma membranes. **d** Mucosa shows a pseudostratified columnar arrangement and the cells display electron-dense granules on the apical side (black arrow). **e** Serosa cells show a cuboidal or squamous shape

Ultrastructural observations in the mesenchyme of the submucosa revealed cell types with undifferentiated morphology: a mainly euchromatic nucleus that occupied most of the cytoplasmic space with none or few small electron-dense granules (Fig. 5). Although several cells in the submucosa showed an undifferentiated morphology (Fig. 5a), we also observed cells that showed features of differentiation including more condensed nuclear chromatin, a smaller nucleus in relation to the cytoplasmic volume, a more irregular plasma membrane, and a higher amount of larger dense vesicles or vacuoles in the cytoplasm (Fig. 5a, b). Cells with undifferentiated morphology were generally observed as clusters and were often found to be associated with glandular cell types (Fig. 5b). In Fig. 5b, part of a gland cell in the submucosa is shown beside a small cell with a big nucleus and thin cytoplasm, but most importantly with a euchromatic nucleus and nucleoli, which are the characteristic features of hemoblast cells. Submucosa cell aggregates also contained cells that showed early and late features of differentiation within the same aggregates. Some of these cells were glandular (Fig. 5b), or presented irregular plasma membranes and lower nuclear/cytoplasm ratios (features of differentiation), and other larger cells showed distinct cytoplasmic granules of differentiated granulocytes (Fig. 5c). Cells in the mucosa showed a typical columnar disposition, with electron-dense granules mainly concentrated on the apical side of each epithelial cell (Fig. 5d). In contrast, cells in the serosa epithelium presented a more cuboidal shape (Fig. 5e).

### *Styela plicata* circulating cells are first apparent during metamorphosis

**Fig. 6:**
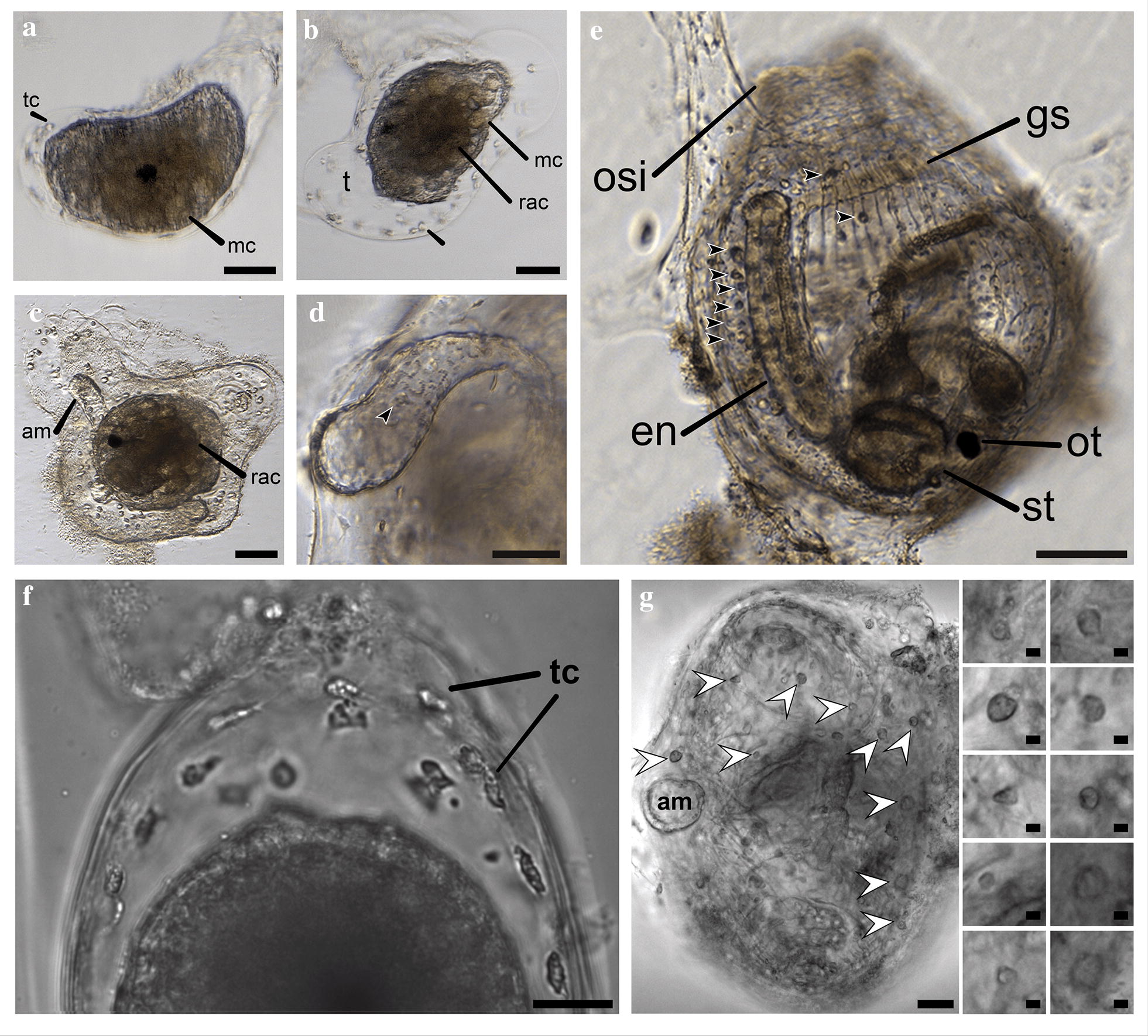
Motile cells become apparent during metamorphosis. **a** Early stage of metamorphosis, 17 h30 m post-fertilization, shows the earliest motile cells: ameboid tunic cells and globular mesenchyme cells (pf); **b** mid-metamorphosis stage, 20 h 30 m pf, reveals the rudiment of the alimentary (rac) canal in differentiation; **c** late metamorphosis stage, 25 h 15 m pf, exhibits the presence of extended ampullae; **d** detail of a post-metamorphic ampulla, 48 h pf, reveals new circulatory cell types in the lumen (arrowhead); **e** juvenile, 48 h pf, indicates the presence of pigmented circulating cells (arrowheads). **f** Detail of amoeboid tunic cells during metamorphic expansion of the tunic. **g** Detail of circulating cells (arrowheads and insets on the right) in a ventral view of a juvenile. Tunic cell (tc), globular mesenchyme cell (mc), tunic (t), otolith (ot), rudiment of the alimentary canal (rac), ampulla (am), oral siphon (osi), endostyle (en), gill slit epithelium (gs), stomach (st). Scale bars = 100 µm in **a**–**e**; 50 µm in **f**; 25 µm in **g** (left), 5 µm in **g** (right)

The first sign of freely moving cells during development of *S. plicata* occurred during metamorphosis. As the tadpole larval tail began to resorb during the first phase of metamorphosis, notochordal cells contracted and posterior muscle cells in the tail lost adhesion and underwent morphogenetic changes. At this stage, we observed large actively moving round brown cells, here referred to as globular mesenchyme cells (mc), which migrated actively towards the tadpole head (Fig. 6). Completion of larval tail resorption and exclusion of the larval tunic occurred at about 8 h after tail resorption began. At this time, the mesenchyme cells that had initiated migration from the posteriormost structures of the tail were now localized on the anterior part of the former larval head and surrounded the rudiment of the alimentary canal (Fig. 6a, b).

After 8 h post tail resorption, a second phase of metamorphosis began, in which the tunic expanded on the substrate and the juvenile differentiated. At this time, amoeboid cells were observed crawling into the larval tunic in exclusion, and became more active as the juvenile tunic expanded (Fig. 6a, b, f). At this stage, four protrusions derived from the epidermis—very similar to colonial ascidian ampullae— began to extend radially and ventrally in four directions from the animal (Fig. 6b, c), and during the next 2–5 h, the ampullae reached full extension (Fig. 6c, d). Ampullae either extended on the underlying substrate or in the absence of a substrate they extended outwards or upwards, i.e., distally from the body. At this stage, the ampullae began to exhibit circulating cells in their lumen at the same time as waves of contractions of the ampullar epithelia were observed. Each wave lasted approximately 1 min (data not shown).

After 1 week of settlement, the four juvenile ampullae were resorbed, and the tunic of the juvenile continued to thicken. Simultaneously, the siphons opened and the cilia in the gill slits of the branchial basket started to beat. Shortly after these events, the first heartbeats were observed. Hemocytes with heterogeneous morphologies and sizes were already present at this stage (Fig. 6e, g). Thus, by the first week after fertilization, the circulatory system was clearly functional.

## Discussion

### Circulating putative stem cells (CPSCs) in solitary and colonial styelid ascidians

In the solitary styelid *Styela plicata,* we observed two cell types that show a small size, round shape, and large nucleus: hemoblasts and lymphocyte-like cells. The slightly larger hemoblast (~ 6 µm) contains one or several nucleoli in its nucleus and represents the undifferentiated hemocyte type or CPSC; whereas, the lymphocyte-like cell is slightly smaller (~ 3–5 µm), lacks nucleoli and has been implicated in inflammatory reactions [25, 47]. In contrast, in the *Botryllus* literature the term ‘lymphocyte-like cell’ or simply ‘lymphocyte’ has been misused to refer to the hemoblast or the undifferentiated circulating hemocyte type [48]. The two terms to describe a single cell type in *Botryllus*, as well as the presence of two morphologically similar cell types in *Styela*, have generated some confusion in the nomenclature of progenitor hemocyte types in ascidians. Although further comparative and functional studies of the lymphocyte-like cells and hemoblasts in different species of *Styela* are needed to unequivocally distinguish these two hemocyte types, microscopic observations have raised the possibilities that these cells correspond to immature or transitional stages of the same cell type [5, 49, 50], where hemoblasts may be the general precursor stem cells giving rise to lymphocyte-like cells and other hemocytes (unpublished observations from our labs), or alternatively that these cells represent different cell type progenitors, i.e., immunologically active undifferentiated cells and hematopoietic cells, respectively [5, 49, 51]. Likely biased by an equivocal distinction between lymphocyte-like cells and hemoblasts, Sawada et al. [52] reported the presence of one single small hemocyte type (4–6 µm in diameter) that represented ~ 14% of all cell types in the hemolymph. This size range and proportion of small cells in the hemolymph of *Styela clava* are very similar to the size range and proportion of small hemocytes we report here for *S. plicata* (3–6 µm in diameter and ~ 16.7%) and likely correspond to both small cell types combined, i.e., lymphocyte-like cells + hemoblasts. Thus, it is very likely that *S. clava* also contains both types of small cells present in *S. plicata*. In summary, we highlight the occurrence of distinct circulating cell types resembling undifferentiated cells in *S. plicata*, and argue for their occurrence in other solitary *Styela* species.

The existence of CPSCs in the hemolymph of *S. plicata* is further supported by the presence of a low-granularity ALDH + population of hemolymph isolated by flow cytometry. But what is the frequency of cells with low granularity and high ALDH expression in a solitary ascidian when compared to a colonial ascidian? In the colonial *B. schlosseri,* it has been estimated that 1 out of 500–1000 hemocytes corresponds to circulating stem cells that are able to replicate and differentiate inducing genetic chimerism in parabiosed partners [12]. After ALDH enrichment, 1 out of 25–50 high ALDH expressing cells (ALDH +) shows stem cell capabilities, i.e., self-renewal and multilineage potential. Thus, the ALDH assay increases 10–20-fold the frequency of putative stem cells in a population of ALDH + cells when compared to a non-sorted control population of hemocytes. In contrast, the ALDH assays in this study generated a fourfold enrichment of ALDH + hemocytes in *S. plicata*; in other words, ALDH + cells represent 7.36 ± 6.8% of all *S. plicata* hemocytes. A lower enrichment of ALDH + cells observed in our assays may reflect a lower occurrence of CPSCs in *S. plicata* than in *B. schlosseri*. Prospective isolation of ALDH + cell populations, as well as non-sorted hemocyte populations, is necessary to better estimate the fractions that correspond to CPSCs in the *S. plicata* hemolymph. In sharp contrast, we estimated in direct visual cell counts that ~ 17% of all hemocytes presented morphological features of CPSCs in *S. plicata*; whereas, only ~ 4% of all hemocytes correspond to hemoblasts in *B. schlosseri* and *Polyandrocarpa misakiensis* [23, 53]. The difference between the percentages of ALDH + cells and hemocytes with a CPSC morphology between the visual counts and the flow cytometry counts in *S. plicata* may be due to the fact that both hemoblasts and lymphocyte-like cells were counted together under the category of small cells because in many cases we could not tell them apart. It remains to be determined what fraction of the small cells corresponds exclusively to CPSCs.

When we compare all the different hemocytes that have been identified in Styelidae, we find that circulating cell types have been gained and lost in the different styelid species [5, 54], but overall we observe the occurrence of more kinds of hemocytes in colonial styelids than in their solitary counterparts. Nonetheless, *S. plicata* contains two hemocytes (i.e., hemoblasts and lymphocyte-like cells) that resemble CPSC morphology, while all colonial styelid species—including *Botryllus* spp., *Botrylloides* spp., *P. misakiensis,* and *Symplegma brakenhielmi*—studied to date only show the occurrence of the hemoblast cell type. Thus far, we have not found any evidence for the occurrence of a *Styela*-like lymphocyte lineage (see above) in any colonial styelid species [23, 28, 53, 54]. In general, the presence of more kinds of hemocytes in colonial ascidians implies that these cells have specialized in relation to biological processes of a colonial life history, such as allorecognition, colony homeostasis, colony-wide processes development, or asexual reproduction.

### The *Styela plicata* intestinal submucosa as a putative stem cell niche

The intestinal submucosa cell types and general organization in *S. plicata* show resemblance to previously described stem cell “niches” in other animals [1, 55, 56]. In adult fish, for example, a heterogeneous population of mature hematopoietic cells and their precursors are observed among fat and stromal elements in compartments situated between the renal tubes of the kidney [55]. Both by histology and ultrastructure, precursor cells in the hematopoietic compartments of fish [55, 57] were very similar to the undifferentiated cell progenitors we observed in the aggregates in the intestinal submucosa of *S. plicata*.

Our data show the occurrence of a population of undifferentiated cells or putative hemoblasts confined within an extracellular matrix in the intestinal submucosa. As observed, in many animal species, such as crustaceans, mammals, and *Drosophila* [58–60]. The fate of CPSCs is regulated by a particular microenvironment, known as a “niche”. Studies have revealed that the niche corresponds to a specific localization of the body where stem cells reside [61]. The niche functions as a physical anchor for stem cells, apart from generating extrinsic or intrinsic factors that control their number and fate [62]. In many types of niches, the basement membrane and extracellular matrix of niche epithelial cells provide mechanical or signaling anchoring for stem cells [62, 63]. In the *S. plicata* gut, we observed several aggregates of undifferentiated cells within the extracellular matrix of the submucosa region in close proximity to the basement membranes of either the mucosa or serosa epithelial cells. Such an organization of hemoblast aggregates in the intestinal niche suggests the possible presence of *paracrine* signaling from the basement membranes of the intestine and/or *autocrine s*ignaling from the hemoblasts themselves.

Cellular aggregates in the *S. plicata* intestinal submucosa show similarities to putative stem cell niches that have been documented for other ascidians, including *Styela clava* [49] and *Ciona* [64, 65]. Ermak [49, 66] observed in various styelids that stem cell niches are either organized in nodules or appear diffused. In *S. clava*, he localized hematopoietic nodules along the transverse and longitudinal vessels of the pharynx, on the body wall endocarps and around the gut and gonads [49, 67]. The pharyngeal nodules of *S. clava* also contained clusters of hemoblasts surrounded by various cell types at different stages of differentiation, as we described for the intestinal submucosa of *S. plicata* in this study; however, hemocyte differentiation follows a more organized, sequential, and concentric arrangement of hemocyte differentiation in the pharyngeal nodules of *S. clava* [50] in sharp contrast to the less organized arrangement observed in cell aggregates of the intestinal submucosa of *S. plicata*. The different morphological arrangements of these putative niches in *S. clava a*nd *S. plicata* suggest a clearly distinct cellular and developmental nature of these tissues. In contrast in colonial styelids—specifically in *Botryllus* and *Botrylloides*—several potential hematopoietic nodules and stem cell niche locations have been proposed, including: (i) in the anterior ventral region of the endostyle [68], (ii) in ventrally located cellular aggregates known as cell islands [69], (iii) in cellular aggregates lining within the vessels of the circulatory system of colonies [49, 70], and (iv) in compact cell clusters of the gonads [71]. Altogether, these results suggest the presence of multiple adult niches in the different styelid species, which may be specialized to contain different progenitors.

To test whether putative hemoblasts in the intestinal submucosa of *S. plicata* showed a molecular profile of stem cells, we analyzed molecular markers of stemness (PIWI) and proliferation (pHH3). PIWI belongs to the PIWI/Argonaute superfamily of RNA interference effector proteins, and is essential for self-renewal and maintenance of germline and somatic stem cells [15, 20, 72, 73]. As expected, we observed that PIWI + cells in the ovary (Additional file 3: Figure S2), but were also well represented in the submucosa of the *S. plicata* intestine (approx. 17% of all cell types), from which only a third showed pHH3 expression (approx. 6%). Although most pHH3 + cells were small and resembled hemoblast-like cells, PIWI + cells varied significantly in size and shape. Studies of PIWI expression in the *Drosophila melanogaster* ovary have shown that PIWI is expressed not only in germline stem cells (GSCs) to maintain undifferentiation, but also in two other differentiated somatic cell types of the ovary: (a) the associated somatic cap cells that also contribute to the maintenance of undifferentiation in GSCs, and (b) in the escort cells that actually promote GSCs differentiation [74]. Therefore, PIWI expression in multiple differentiated cell types of the *S. plicata* intestinal submucosa is not entirely unexpected, and it is tantalizing to consider that these cells have a role in the regulation of hemoblast maintenance or differentiation, as shown above for PIWI-expressing somatic cells in the *Drosophila* ovary. As an alternative, the commercially available antibody used in this study may be recognizing two distinct PIWI isoforms that may be expressed in different cell types [15], including undifferentiated hemoblasts and other differentiated somatic cells of the *S. plicata* submucosa (Additional file 3: Figure S2). At present, we believe that the true population of hemoblasts in the submucosa consists of a subset of proliferating small cells that simultaneously express PIWI (< 5% of cells present in the submucosa). To reliably identify and calculate the occurrence of hemoblast populations in the *S. plicata* submucosa, future studies should clone a handful of stem cell marker genes in *S. plicata* to test for co-expression of these markers in single progenitor and precursor cells of the submucosa.

Are hemoblasts in the submucosa able to transmigrate through the serosa epithelia to become CPSCs in the hemocoel in *S. plicata*? Although the undifferentiated cells in the intestine resemble hemoblasts in the hemolymph in their morphology [25] (i.e., a high nucleus/cytoplasm ratio, presence of nucleoli within the large nucleus, and presence of small electron-dense granules with few or no organelles in the cytoplasm), our data do not show a developmental or functional relationship between these two populations. Some of us are currently testing the potential of progenitor cells in the submucosa, including the hemoblasts, as a source of CPSCs in the hemolymph by cell culture, transplant, and tracking analyses.

### Ontogenetic origin of circulatory cells occurs at metamorphosis

To explore the ontogenetic origin of adult circulatory putative stem cells (CPSCs) in *Styela plicata*, we characterized cell types and observed cell behavior during metamorphosis and early juvenile development. In vertebrates, circulatory cells of the blood originate in four sequential waves, in which the distinct hematopoietic cell lineages arise temporally apart from each other. The development of embryonic (i) macrophages and (ii) erythrocytes represent the first two waves during the so called “primitive” hematopoiesis, whereas the development of (iii) erythro-myeloid progenitors (EMPs) and (iv) multipotent hematopoietic stem cells (HSCs) represent the last two waves of the “definitive” or adult hematopoiesis in vertebrates [75]. In this first attempt to characterize the earliest appearance of circulatory cells (i.e., hemocytes) in *S. plicata*, we could observe the occurrence of at least three sequential waves. We hypothesize that the first two waves correspond to the appearance of the (i) amoeboid cells and (ii) globular mesenchyme cells during the early events of metamorphosis, and which may be considered analogous to the “primitive” hematopoiesis of vertebrates as these mesenchymal and freely moving hemocytes appeared only during metamorphosis. Although we could not identify the origin or molecularly characterize any of these early motile cell types in *S. plicata*, we suspect that the globular mesenchyme cells correspond to apoptotic muscle cells that dissociate during tail resorption previously observed in other metamorphosing solitary ascidians [76, 77]. The following sequential waves of hematopoiesis only occurred a week later (1 week after settlement) and gave rise to other various hemocytes, including hemoblast precursors or CPSCs of juveniles and adults. These observations only represent the beginning of more research that is necessary to reliably elucidate the developmental origin of each adult hematopoietic lineage.

In contrast to the characteristic waves of hemocytes and circulatory cells that arise during ontogeny, adult development rarely generates any new hemocyte types [78]. Although we did not observe a change in the number of circulating cell populations in *S. plicata* adults of different sizes, we observed increased variation of granularity in hemocytes of larger animals consistently. Recent studies in human cell lines have shown a relationship between granularity in cells (estimated by SSC) and cellular senescence [79, 80], which results from the accumulation of more lysosomes and other autophagic vesicles [81]. According to Yamaguchi [82], *S. plicata* individuals of approximately 40 mm are already sexually mature, and can be between 2 months (summer) or 5 months (winter) old. Because ascidians in our study ranged between 10 and 45 mm, we can estimate the age of the ascidians used here to be between 1 and 5 months old [82]. Although we favor the hypothesis that more variation in granularity in hemocytes of larger *S. plicata* individuals is a result of a senescent phenotype, we cannot exclude the possibility that as animals grow, the diversity of granulated hemocytes increases due to physiological or developmental demands. It may be interesting to compare the ontogenetic diversification of hemocytes, as well as hemocyte senescence in solitary and colonial ascidians where demands of particular types of hemocytes change during the life cycle.

### Implications of CPSCs for the evolution of coloniality in styelids

The presence of CPSCs in Styelidae raises questions about their developmental dynamics, and possible roles in developmental processes. Whereas CPSCs in colonial styelids, including *Botryllus* and *Botrylloides*, have been studied for their involvement in asexual reproduction and regeneration, the presence of CPSCs with hemoblast features (i.e., lack of granules, round morphology with prominent nucleus, and high ALDH activity) in the solitary *Styela* is not at all surprising because of its well-documented siphon and neural complex regeneration abilities [27, 51, 67]. Thus, the presence of CPSCs in all solitary and colonial ascidians studied so far supports the hypothesis that the styelid ancestor likely contained CPSCs and mechanisms to regulate CPSC functional dynamics. We currently have evidence for the evolution of at least two independent events of coloniality in the Styelidae [21], suggesting a degree of evolvability in the developmental program of adult stem cell lineages. Key modifications in adult stem cell characteristics may have allowed the developmental processes underlying coloniality to evolve as a result of novel selective interplays between cellular lineages. Further insights into this evolutionary transition will come from comparisons among species of interest in Styelidae.

## Conclusion

We report the presence of morphologically undifferentiated cell types with high aldehyde dehydrogenase activity in the hemolymph of the solitary ascidian *Styela plicata*, which we refer to as circulating putative stem cells (CPSCs). In adult individuals, we find mitotic undifferentiated cell aggregates in the intestinal submucosa that resemble a hematopoietic niche. The earliest appearance of hemocytes during development occurs during metamorphosis of the larva, at a time when circulatory mesenchyme cells show distinct morphotypes from juvenile or adult hemocyte populations. We highlight the importance of our findings for future investigations about the direct involvement of CPSCs in regeneration of solitary ascidians, and about the consequences of the presence of CPSCs in styelid ascidians for the evolution of coloniality.

## Supplementary information

**Additional file 1: Figure S1.** ALDEFLUOR^TM^ analyses optimization procedures for excluding doublets and debris, and to evaluate autofluorescence of *Styela plicata* hemocytes. (A) Gating strategy used to exclude doublets and debris from the analysis. The scatterplots present all data points available. The gate ‘single cells’ (Red) was selected based on area and aspect ratio features (width in relation to total area) and using direct observations of single celled images for further analysis. (B) Scatterplot of the ‘single cells’ gate showing one possible gating strategy to separate ‘small’ and ‘large cells’. (C) Histograms showing the normal frequencies of cells at different intensities of fluorescence in the green channel (Ch02) of the ‘small cells’ and ‘large cells’ gates in the blank control above (i.e., no BAAA), and in cells treated with BAAA (i.e., ALDEFLUOR^TM^); the dotted line indicates the recommended threshold of intensity to consider a positive result. (D) Randomly selected brightfield and fluorescent images of cells of the ‘small cells’ and ‘large cells’ gates of the blank control show complete absence of autofluorescence. (C, D) demonstrate a clear absence of autofluorescence in *S. plicata* hemocytes.

**Additional file 2: Table S1.** Gate polygon coordinates for *Styela plicata* hemocyte BAAA assay.

**Additional file 3: Figure S2.** Evaluation of PIWI antibody cross-reactivity. (A) Alignment of PIWI proteins in the fly *Drosophila melanogaster* (Dromel), the annelid *Pristina leidyi* (Prilei), and ascidians: *Styela plicata* (Stypli), *Botrylloides leachi* (Botlea), *Botryllus primigenus* (Botpri), *Botryllus schlosseri* (Botsch), and *Ciona robusta* (Ciorob). Conserved PAZ and PIWI domains are shown below the *Drosophila melanogaster* PIWI sequence (Dromel_piwi_varA). The sequence region used to generate the epitope for the commercially available antibody used in this study is also shown overlapping the PAZ domain (582–689 aa). Color codes used to represent sequence conservation (Blosum62 score matrix for similarities): white (< 60%), light gray (60–79%), dark gray (80–99%), and black (100%). Sequence similarities (Blosum62) at the region of the epitopes between *S. plicata* and *D. melanogaster* PIWI orthologs are above 70% (i.e., 70.1% Stypli_piwi_b vs. Dromel_varA and Dromel_varB; and 72.5% Stypli_piwi_a vs. Dromel_varA and Dromel_varB). (B–D) Positive control shows PIWI + cells (arrows) surrounding three oocytes of different stages (asterisks); DAPI in (B), 1ary anti-PIWI + 2ary Alexa488 in (C), and overlay in (D); Note: Because PIWI is expressed in the germ cells of ovaries in all ascidian species studied to date, we used positive cell labelings in presumptive germ cells in *S. plicata* ovaries as indicative of PIWI expression. (F–H) Blank control (i.e., no 1ary anti-PIWI antibody) of *S. plicata* ovaries shows absence of labeling in germ cells around the oocytes; DAPI in (B), only 2ary Alexa488 in (C), and overlay in (D).

## Data Availability

Data sharing not applicable to this article as no datasets were generated or analyzed during the current study
