# Supplementary figures and images for: Putative stem cells in the hemolymph and in the intestinal submucosa of the solitary ascidian Styela plicata
Source: EvoDevo. 2019 Nov 25;10:31. doi: 10.1186/s13227-019-0144-3 (PMC6876114; doi:10.1186/s13227-019-0144-3)

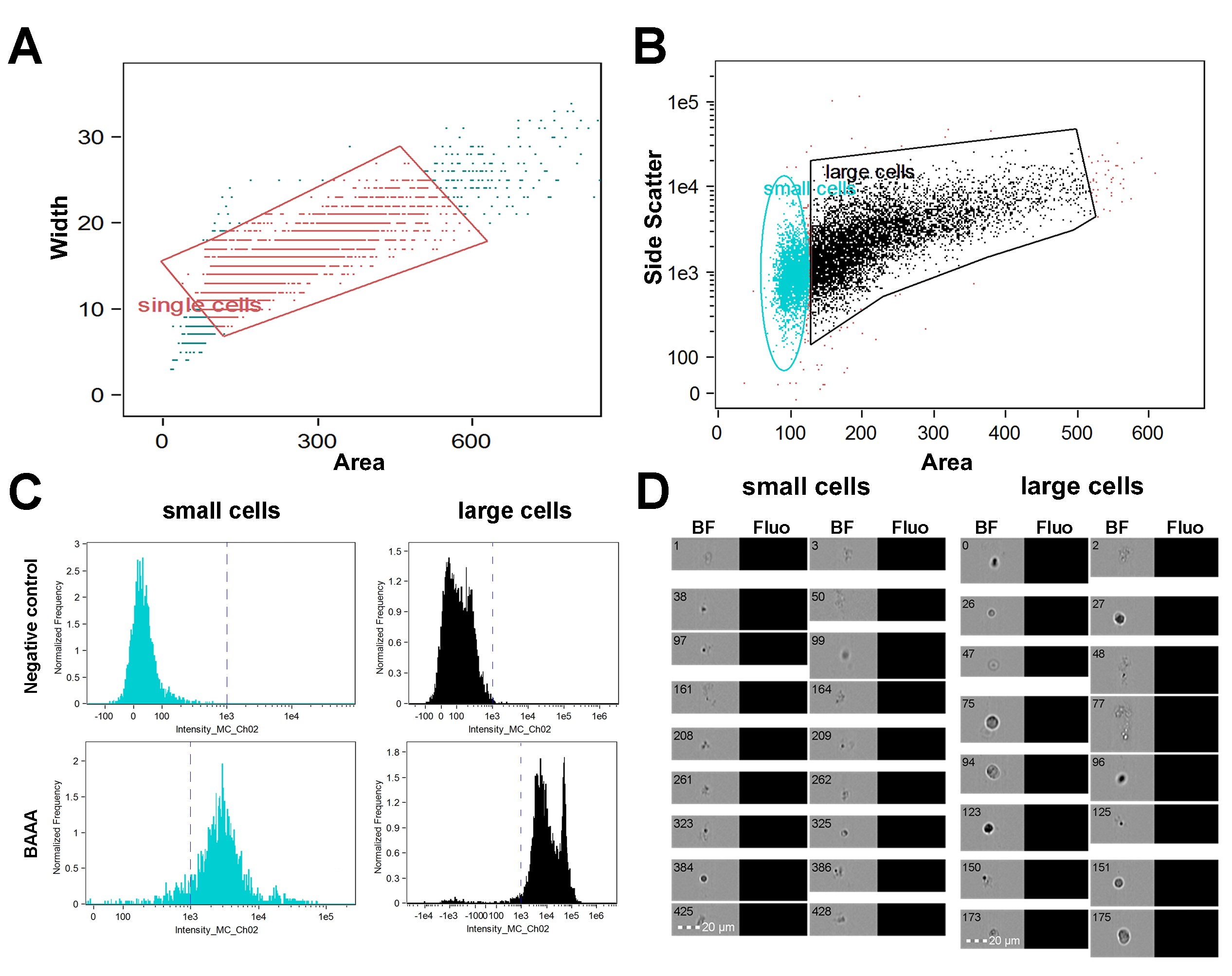

Supplement: Supplementary file 1 — Additional file 1: Figure S1. ALDEFLUORTM analyses optimization procedures for excluding doublets and debris, and to evaluate autofluorescence of Styela plicata hemocytes. (A) Gating strategy used to exclude doublets and debris from the analysis. The scatterplots present all data points available. The gate ‘single cells’ (Red) was selected based on area and aspect ratio features (width in relation to total area) and using direct observations of single celled images for further analysis. (B) Scatterplot of the ‘single cells’ gate showing one possible gating strategy to separate ‘small’ and ‘large cells’. (C) Histograms showing the normal frequencies of cells at different intensities of fluorescence in the green channel (Ch02) of the ‘small cells’ and ‘large cells’ gates in the blank control above (i.e., no BAAA), and in cells treated with BAAA (i.e., ALDEFLUORTM); the dotted line indicates the recommended threshold of intensity to consider a positive result. (D) Randomly selected brightfield and fluorescent images of cells of the ‘small cells’ and ‘large cells’ gates of the blank control show complete absence of autofluorescence. (C, D) demonstrate a clear absence of autofluorescence in S. plicata hemocytes. [file 13227_2019_144_MOESM1_ESM.png]

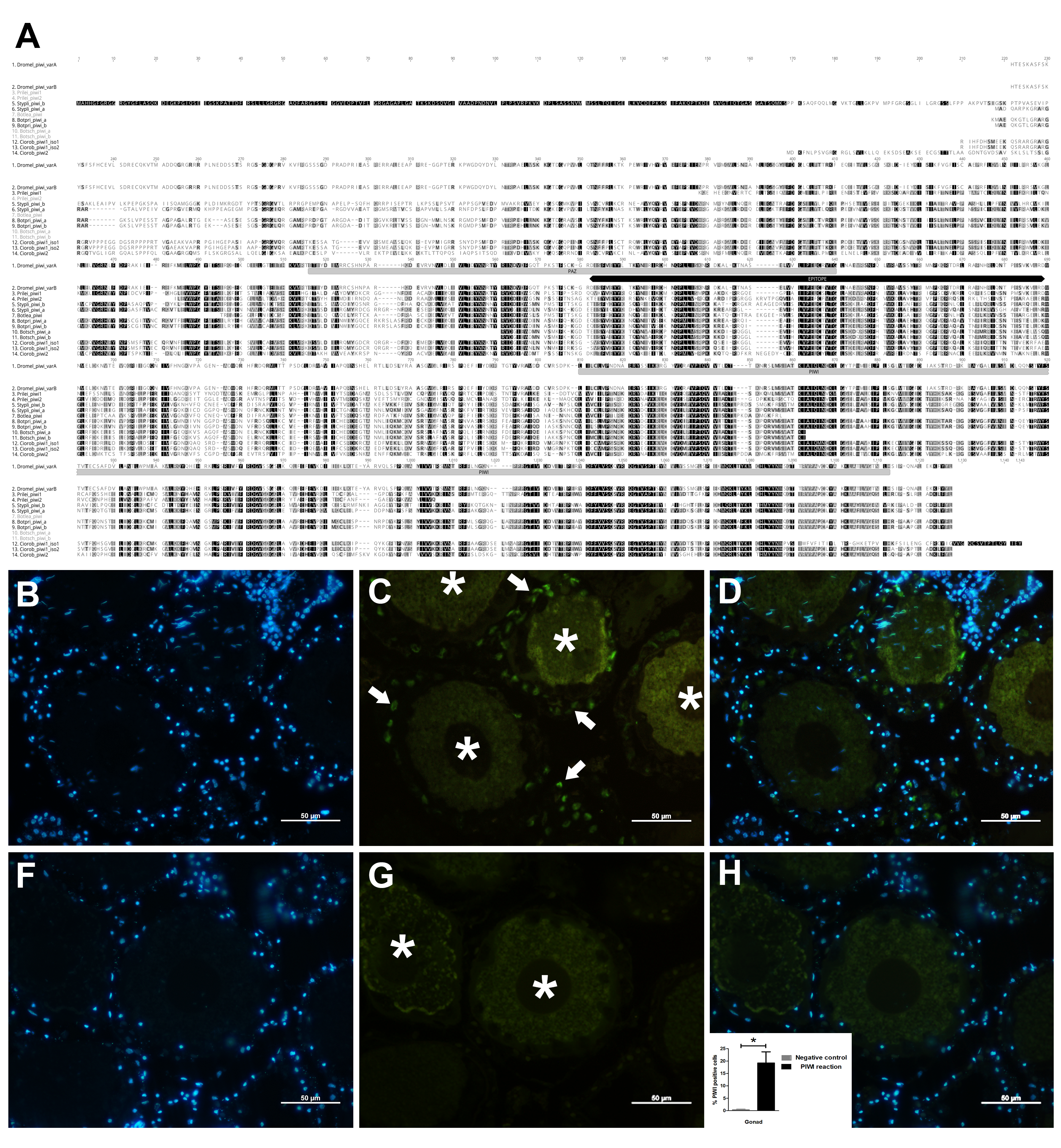

Supplement: Supplementary file 3 — Additional file 3: Figure S2. Evaluation of PIWI antibody cross-reactivity. (A) Alignment of PIWI proteins in the fly Drosophila melanogaster (Dromel), the annelid Pristina leidyi (Prilei), and ascidians: Styela plicata (Stypli), Botrylloides leachi (Botlea), Botryllus primigenus (Botpri), Botryllus schlosseri (Botsch), and Ciona robusta (Ciorob). Conserved PAZ and PIWI domains are shown below the Drosophila melanogaster PIWI sequence (Dromel_piwi_varA). The sequence region used to generate the epitope for the commercially available antibody used in this study is also shown overlapping the PAZ domain (582–689 aa). Color codes used to represent sequence conservation (Blosum62 score matrix for similarities): white (< 60%), light gray (60–79%), dark gray (80–99%), and black (100%). Sequence similarities (Blosum62) at the region of the epitopes between S. plicata and D. melanogaster PIWI orthologs are above 70% (i.e., 70.1% Stypli_piwi_b vs. Dromel_varA and Dromel_varB; and 72.5% Stypli_piwi_a vs. Dromel_varA and Dromel_varB). (B–D) Positive control shows PIWI + cells (arrows) surrounding three oocytes of different stages (asterisks); DAPI in (B), 1ary anti-PIWI + 2ary Alexa488 in (C), and overlay in (D); Note: Because PIWI is expressed in the germ cells of ovaries in all ascidian species studied to date, we used positive cell labelings in presumptive germ cells in S. plicata ovaries as indicative of PIWI expression. (F–H) Blank control (i.e., no 1ary anti-PIWI antibody) of S. plicata ovaries shows absence of labeling in germ cells around the oocytes; DAPI in (B), only 2ary Alexa488 in (C), and overlay in (D). [file 13227_2019_144_MOESM3_ESM.png]
