# Supplementary material for: Putative stem cells in the hemolymph and in the intestinal submucosa of the solitary ascidian Styela plicata
Source: EvoDevo. 2019 Nov 25;10:31. doi: 10.1186/s13227-019-0144-3 (PMC6876114; doi:10.1186/s13227-019-0144-3)
Supplement: Supplementary file 2 — Additional file 2: Table S1. Gate polygon coordinates for Styela plicata hemocyte BAAA assay. [file 13227_2019_144_MOESM2_ESM.docx]

##

| **Additional Table S1 - Gate polygon coordinates for *Styela plicata* hemocyte BAAA assay** | | | | | |
| --- | --- | --- | --- | --- | --- |
| **Number** | **Intensity Ch 2** | **Intensity Ch 6** | **Number** | **Intensity Ch 2** | **Intensity Ch 6** |
| Gate 1 | -24 | 608 | Gate 4 | 3087 | 6266 |
|  | -41 | 163 |  | 3122 | 131464 |
|  | -27 | 42 |  | 22919 | 239608 |
|  | 26 | -19 |  | 69700 | 105599 |
|  | 122 | -49 |  | 99031 | 55501 |
|  | 339 | 32 |  |  |  |
| Gate 2 | 61 | 3080 | Gate 5 | 3151 | 6592 |
|  | 348 | 32 |  | 3151 | 3409 |
|  | 1594 | 126 |  | 295800 | 3241 |
|  | 224 | 17283 |  | 193583 | 20123 |
|  |  |  |  | 99037 | 52756 |
| Gate 3 | 225 | 17283 | Gate 6 | 3215 | 3241 |
|  | 1602 | 133 |  | 3215 | 270 |
|  | 3122 | 257 |  | 289758 | 270 |
|  | 3122 | 131464 |  | 289758 | 3241 |
|  | 1003 | 79160 |  |  |  |
